# Proteomic characterization of Omicron SARS-CoV-2 host response

**DOI:** 10.1038/s41421-022-00418-x

**Published:** 2022-05-18

**Authors:** Jianfeng Bao, Rui Sun, Jingwen Ai, Liujia Qian, Fang Liu, Hongyu Wang, Lingling Tan, Xue Cai, Yingqiu Shi, Xiao Liang, Weigang Ge, Jing Wu, Chen Chen, Wenhong Zhang, Jinsong Huang, Tiannan Guo

**Affiliations:** 1 Department of Hepatology, Affiliated Hangzhou Xixi Hospital, Zhejiang University School of Medicine, Hangzhou, Zhejiang China; 2 Westlake Laboratory of Life Sciences and Biomedicine, Key Laboratory of Structural Biology of Zhejiang Province, School of Life Sciences, Westlake University, Hangzhou, Zhejiang China; 3 Institute of Basic Medical Sciences, Westlake Institute for Advanced Study, Hangzhou, Zhejiang China; 4 Center for Infectious Disease Research, Westlake Laboratory of Life Sciences and Biomedicine, Hangzhou, Zhejiang China; 5 Department of Infectious Diseases, National Medical Center for Infectious Diseases, National Clinical Research Center for Aging and Medicine, Shanghai Key Laboratory of Infectious Diseases and Biosafety Emergency Response, Huashan Hospital, Fudan University, Shanghai, China; 6 Institute of Hepatology and Epidemiology, Affiliated Hangzhou Xixi Hospital, Zhejiang University School of Medicine, Hangzhou, Zhejiang China; 7 Westlake Omics (Hangzhou) Biotechnology Co., Ltd, Hangzhou, Zhejiang China

**Keywords:** Proteomic analysis, Innate immunity

Dear Editor,

Since its first reported case in November 2021 in South Africa^1^, the novel SARS-CoV-2 variant Omicron infection has swept across the world. The Omicron variant has over 30 mutations in the spike (S) protein which increase its affinity for ACE2 but decrease the cleavage efficiency of S protein by TMPRSS2, leading to suppressed virulence and reduced replication^2^. Furthermore, a population-scale meta-analysis showed that Omicron led to a significantly lower proportion of clinically severe cases^3^ despite higher transmissibility compared to other variants^4^. However, the underlying nature of host responses to Omicron is not well characterized. Mass spectrometry (MS)-based proteomics enables the systematic investigation of circulating and tissue proteins which sheds light on host responses to SARS-CoV-2 infection^5–8^. Here, we report the characteristic proteome profile of blood samples from patients with Omicron infection.

We enrolled 17 individuals infected with Omicron SARS-CoV-2, six infected with the prototype strain, and one infected with Delta. We also included 29 individuals infected by non-COVID-19 respiratory virus and 14 healthy controls (Supplementary Tables S1, S2). These subjects were matched for age, gender, and symptoms. In the Omicron group, 15 of 17 patients had received up to four doses of inactivated COVID-19 vaccines. No patients in the non-Omicron COVID-19 group and non-COVID-19 respiratory virus infection group had been vaccinated with any COVID-19 vaccine (Supplementary Table S1). Detailed clinical information of all study subjects is provided in Supplementary Table S2.

We collected 53 blood samples from patients with Omicron infection, non-Omicron SARS-CoV-2 infection, and patients with non-COVID-19 respiratory virus infection. This study also included 14 samples from healthy controls before COVID-19 vaccination (hereafter referred to as pre-vaccination samples), paired with 12 post-vaccination samples of the same control subjects (Supplementary Table S2). In total, 90 peptide samples including 11 technical replicates were randomly distributed into six batches (Supplementary Table S2) for tandem mass tag (TMT)-based proteomics analysis, leading to high-quality identification and relative quantification of 1155 proteins (Supplementary Table S3) as evaluated by replicates (Supplementary Fig. S1a, b). As shown in Fig. 1a, the circulating proteomes of Omicron patients were similar to those of the non-Omicron non-severe COVID-19 patients, suggesting that these Omicron cases induced similar host response to those in non-Omicron non-severe COVID-19 cases. The proteomes of healthy controls with and without vaccination were largely different, probably due to enhanced immunity after vaccination. Not surprisingly, their proteomes were also different from those infected with other respiratory viruses. Striking differences were observed among the samples from COVID-19 cases, non-COVID-19 respiratory virus infections, and healthy control cases, suggesting distinct host responses at circulating proteome level against SARS-CoV-2 viruses.

**Fig. 1:**
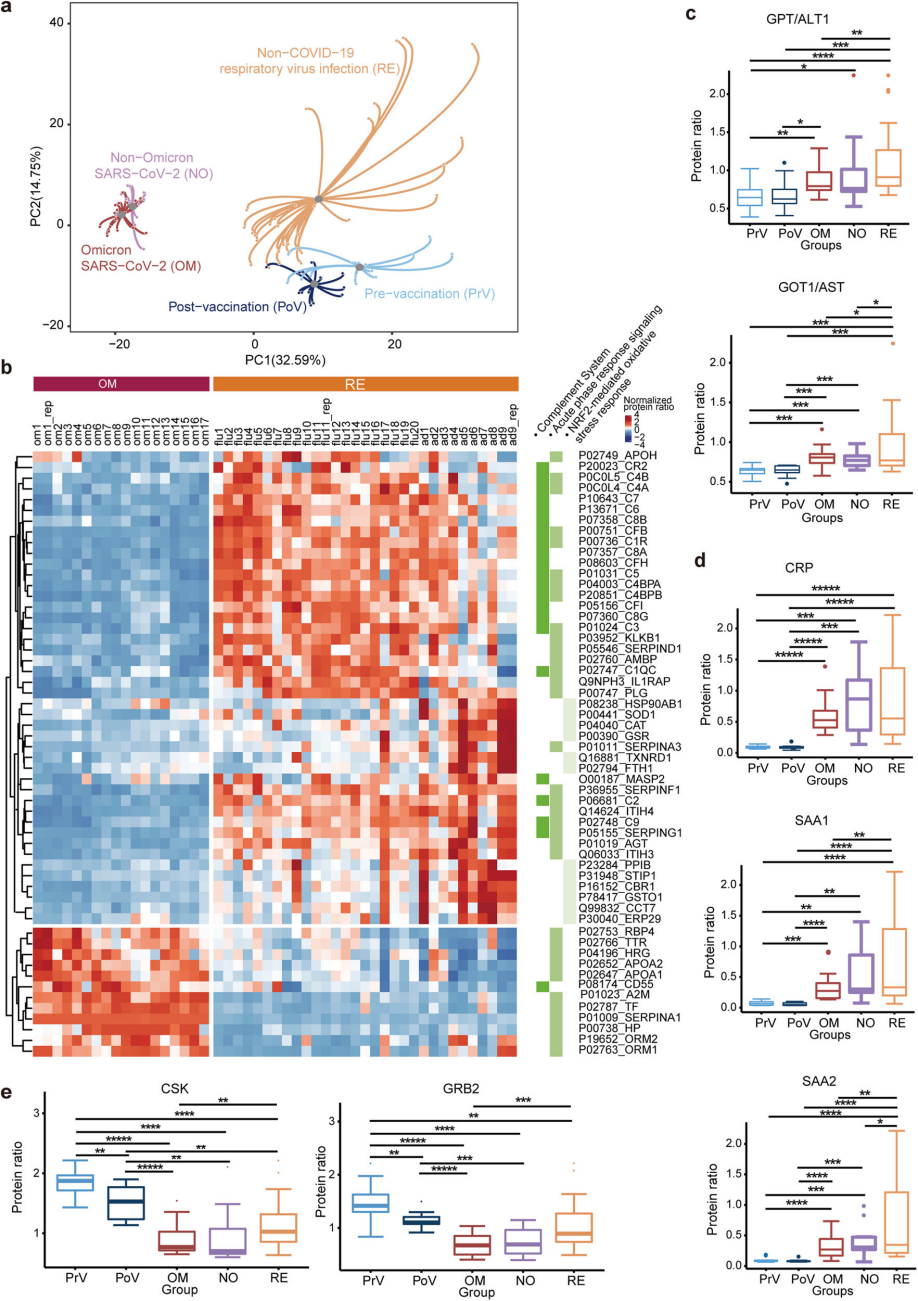
Proteomic analysis of Omicron-induced host responses. **a** Distances of host responses of circulating proteomes in a principal component analysis (PCA) plot. The position of centroid colored in gray is the average value of the PC1 and PC2 coordinates of each sample in a patient group. The line connecting the centroid and each sample represents their distance. **b** 62 differentially expressed proteins between the Omicron and non-COVID-19 groups with fold change > 1.2 and Benjamini–Hochberg (B-H) adjusted *P* < 0.05, as shown in a heatmap. These proteins are enriched in three selected pathways by Ingenuity Pathway Analysis (IPA) (*P* < 0.05). **c**–**e** Differential expression of GPT/ALT1 and GOT1/AST (**c**), CRP, SAA1 and SAA2 (**d**), CSK and GRB2 (**e**) across the five patient groups. PrV, pre-vaccination; PoV, post-vaccination; OM, Omicron; NO, non-Omicron; RE, non-COVID-19 respiratory virus infection. *P < 0.05; **P < 0.01; ***P < 0.001; ****P < 0.0001; *****P < 0.00001.

The Omicron patients showed only two significantly downregulated proteins, namely C2 and SERPING1, compared to the patients with non-Omicron SARS-CoV-2 infection. However, 529 circulating proteins were significantly different between Omicron and non-COVID-19 individuals (Supplementary Table S4), which were enriched in ten pathways (*P* value < 0.05) (Supplementary Fig. S2a). Interestingly, the Omicron specimens showed enriched inflammation-associated pathways including the complement system, acute phase response signaling, and NRF-2 mediated oxidative stress response (Fig. 1b). In addition to characteristic downregulation of complementary proteins, these specimens also exhibited upregulation of acute-phase proteins including histidine rich glycoprotein (HRG), alpha-2-macroglobulin (A2M), serotransferrin (TF), haptoglobin (HP), alpha-1-acid glycoprotein 1 (ORM1) and alpha-1-acid glycoprotein 2 (ORM2), apolipoprotein A-I (APOA1) and apolipoprotein A-II (APOA2) (Fig. 1b). Of note, regulation of these pathways and proteins has also been observed previously when comparing the circulating proteome of non-severe COVID-19 patients with severe cases^5–8^. This suggests attenuated inflammation in the Omicron infections compared with those with other respiratory viruses.

Omicron-induced hepatic injury was comparable to non-Omicron non-severe COVID-19, significantly higher than healthy controls, but significantly lower than that by other respiratory viruses, as evaluated by aspartate aminotransferase (GOT1/AST) and alanine aminotransferase 1 (GPT/ALT1) (Fig. 1c). These observations are supported by several other studies reporting hepatic impairment associated with COVID-19^9^ and influenza virus infections^10^. Injuries of liver can stimulate the release of acute-phase proteins (APPs)^11^. Indeed, we found the elevation of C-reactive protein (CRP), serum amyloid A-1 protein (SAA1), and serum amyloid A-2 protein (SAA2) (Fig. 1d) in the Omicron specimens compared to healthy controls. It has been reported that, compared with non-severe COVID-19 cases, severe cases exhibited hepatic injuries which are associated with elevation of multiple proteins including GOT1/AST, GPT/ALT1, CRP, SAA1, and SAA2^5–8^. The profile of APPs in the Omicron samples followed the known pattern of hepatic injuries, further confirming that Omicron induced comparable liver injuries and inflammation to non-severe COVID-19, which was less intense than flu and flu-like illnesses. Most of the Omicron patients analyzed in this study had been vaccinated, therefore, their attenuated hepatic injuries and immune responses may be partly attributed to vaccination.

Since the Omicron patients received varied doses of vaccination, we investigated whether the doses of vaccination have an impact on the proteome-based host responses. We compared the proteomes of samples from the Omicron patients who had received two doses of vaccination (*n* = 9) with that from the other vaccinated patients (*n* = 6). Two cases without vaccination or with unclear vaccination status were excluded from this analysis. Our data showed no significantly differentially expressed proteins (DEPs). We also performed an analysis of variance (ANOVA) of the Omicron patients who had received 1, 2, 3, and 4 vaccine doses. Neither did we observe any significantly DEPs. Together, these analyses suggest that the variability of vaccination doses did not generate significant variability in host responses in terms of the circulating proteome.

We then compared the circulating proteomes among the 15 samples from Omicron patients with a priori vaccination, as well as 14 pre-vaccination and 12 post-vaccination paired samples from healthy controls. Vaccination downregulated 133 proteins and upregulated one protein in the healthy controls (Supplementary Table S5a), suggesting that these DEPs are associated with enhanced immunity against SARS-CoV-2. Next, we identified 513 DEPs between the Omicron samples and post-vaccination healthy samples (Supplementary Table S5b). Remarkably, 107 proteins which had decreased after vaccination in the healthy controls further declined upon Omicron infection (Supplementary Fig. S2b). These proteins are involved in multiple immune pathways including vesicle-mediated transport, leukocyte-mediated immunity, and complement activation, among others (Supplementary Fig. S2c). A negative regulator of T- and B- cell antigen receptors, namely CSK^12,13^, was downregulated in the Omicron samples (Fig. 1e). Another negative regulator of B-cell receptor antigen-stimulated signaling, GRB2^14^, showed a similar decreasing trend (Fig. 1e). Decreased GRB2 promotes Th17 differentiation and inflammation through MAPK signaling^15^. Interestingly, our data also highlighted the reduction of multiple proteins in the MAPK signaling pathways, including MAPK1, PPP1CA, PPP1R7, PRKACB, PRKAR1A, and PRKAR2B, in Omicron-induced host responses (Supplementary Fig. S2d). Apart from these 107 DEPs, the remaining 406 DEPs between the Omicron and post-vaccination groups were enriched in similar immune pathways (Supplementary Fig. S2e), suggesting that the adaptive immune responses induced by vaccination were further enhanced after Omicron infection.

Despite the limited proteomic data from relatively small sample sets, the experimental design and statistical analysis are reasonable, with conclusions supported by our data. Future analysis of larger cohorts, taking into account more clinical covariates, coupled with mechanistic studies, are needed to systematically investigate the entire landscape of Omicron-induced host responses.

In summary, our proteomic analysis shows that in non-severe cases, Omicron induced similar host responses in vaccinated individuals compared to non-Omicron SARS-CoV-2, which is more intense than those in healthy donors, but less severe than those in flu and flu-like patients. The potential liver injuries of Omicron infections might be weaker than those of other respiratory viruses.

## Supplementary information

Supplementary Information

Supplementary Table S2

Supplementary Table S3

Supplementary Table S4

Supplementary Table S5
