# Supplementary material for: Proteomic characterization of Omicron SARS-CoV-2 host response
Source: Cell Discov. 2022 May 18;8:46. doi: 10.1038/s41421-022-00418-x (PMC9114018; doi:10.1038/s41421-022-00418-x)
Supplement: Supplementary file 1 — Supplementary Information [file 41421_2022_418_MOESM1_ESM.pdf]

Supplementary Information

Materials and Methods

Patient and sample information

The Wuhan-Hu-1, Delta and Omicron SARS-CoV-2 (BA1.1) patients were diagnosed by rapid genome sequencing in portable nanopore sequencers from Xixi Hospital, Hangzhou, China. Fifteen of 17 patients had received inactivated virus vaccines previously. Serum samples were acquired after fasting for 8 hours and were centrifuged at 1500 g for 10 min before storage at -80°C.
Non-COVID-19 respiratory virus infected patients were diagnosed via Xpert® Xpress Flu/RSV Assay or next-generation sequencing between 2015 and 2021 from Huashan Hospital [1]. Healthy participants were enrolled from 2021 to 2022 and all their plasma samples were collected pre-vaccination and 14 days after the second dose of Sinovac [2]. Paired plasma and serum samples were obtained from additional healthy participants. All plasma samples were acquired from EDTA-anticoagulated peripheral blood and were stored at -80°C. Another eight plasma samples and their matching seven serum samples from three individuals were used to adjust for differences between plasma and serum samples. These 15 samples formed an independent analytical batch. Ethical approvals were obtained from the Xixi Hospital, Huashan Hospital and Westlake University.

Proteomics analysis

Serum/plasma samples were inactivated in 56°C for 30 min, followed by depletion of the top 14 high abundance proteins using the High Select™ Top14 Abundant Protein Depletion Resin (Thermo Fisher Scientific ™, San Jose, USA) and proteolytic digestion into peptides as described previously [3]. The clean peptides were labeled with tandem mass tag (TMT)pro and fractionated using Thermo Scientific™ UltiMate™ 3000 RSLC system [3]. Each fraction was analyzed with Orbitrap Exploris 480 as described previously [4]. MS data were analysed using Proteome Discoverer (version 2.5, Thermo Fisher ScientificTM, Waltham, MA) as described previously [4] against a human protein sequence database from UniProt (containing 20,365 reviewed proteins) downloaded on 14, Apr 2020.

Data analysis

1464 proteins were identified from the TMTpro data sets. After filtering out proteins missing in over 80% of the samples, 1155 proteins were identified. The missing values were imputed with sequential k-Nearest Neighbor method [5]. A two-sided unpaired Welch's *t*-test was used for comparisons between two groups. *P* was adjusted by the Benjamini-Hochberg (B-H) method. Taking account of the proteomic differences between plasma and serum, we removed 404 differentially expressed proteins (DEPs) ($P < 0.05$ and fold change > 1.2 or < 0.84) between the serum and

plasma which appeared either in our datasets of plasma and serum samples, or in a published dataset [6]. A total of 751 unique proteins were subject to subsequent analyses.

Pathway analysis

GO biological processes were used for pathway enrichment analysis. Ingenuity Pathway Analysis (IPA, version 51963813) was used to identify pathways enriched in the DEPs. The most significantly enriched pathways had $P < 0.05$ and contained at least five proteins identified in our dataset.

Data availability

All data are available in the manuscript and supplementary materials. The proteomics data are deposited in ProteomeXchange Consortium (https://www.iprox.org/). Project ID: IPX0004146001. All the data will be publicly released upon publication. All the codes used in this study are provided in Github with a link https://github.com/guomics-lab/CVDO

References

1 Qian, Y. Y. *et al.* Improving Pulmonary Infection Diagnosis with Metagenomic Next Generation Sequencing. *Front Cell Infect Microbiol* **10**, 567615 (2020).

2 Lin, K. *et al.* B cell receptor signatures associated with strong and poor SARS-CoV-2 vaccine responses. *Emerg Microbes Infect* **11**, 452-464 (2022).

3 Shen, B. *et al.* Proteomic and Metabolomic Characterization of COVID-19 Patient Sera. *Cell* **182**, 59-72 e15 (2020).

4 Zhang, X. *et al.* sRAGE alleviates SARS-CoV-2-induced pneumonia in hamster. *Signal Transduct Target Ther* **7**, 36 (2022).

5 Kim, K. Y., Kim, B. J. & Yi, G. S. Reuse of imputed data in microarray analysis increases imputation efficiency. *BMC Bioinformatics* **5**, 160 (2004).

6 Geyer, P. E. *et al.* Plasma Proteome Profiling to detect and avoid sample-related biases in biomarker studies. *EMBO Mol Med* **11**, e10427 (2019).

Supplementary figures

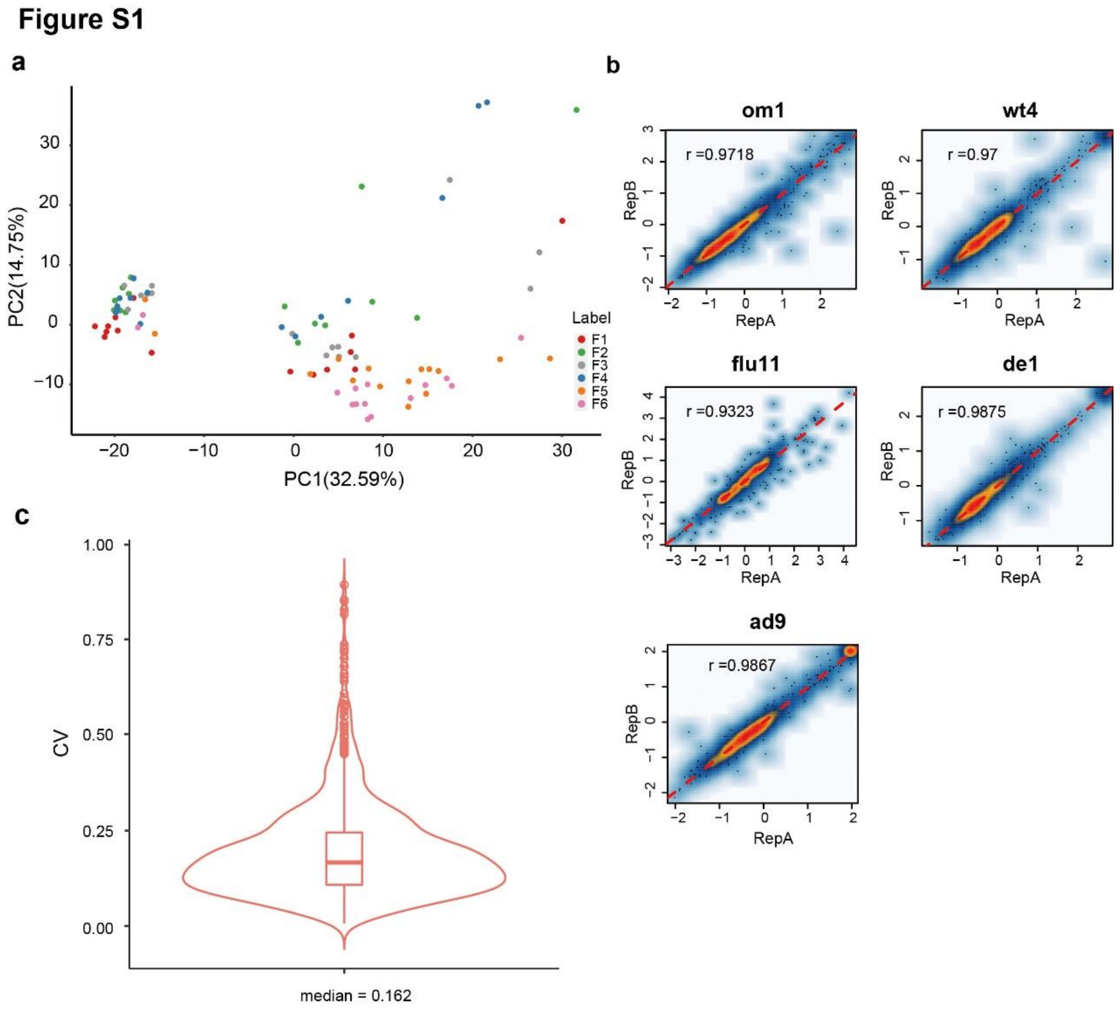

Supplementary Figure 1. Quality control of proteomic data. a principal component analysis (PCA) plot of all samples from multiple batches. **b** Correlation analysis between the technical replicates. **c** Violin plot showing the coefficients of variation (CV) of the pooled samples for data normalization across different batches.

Figure S2

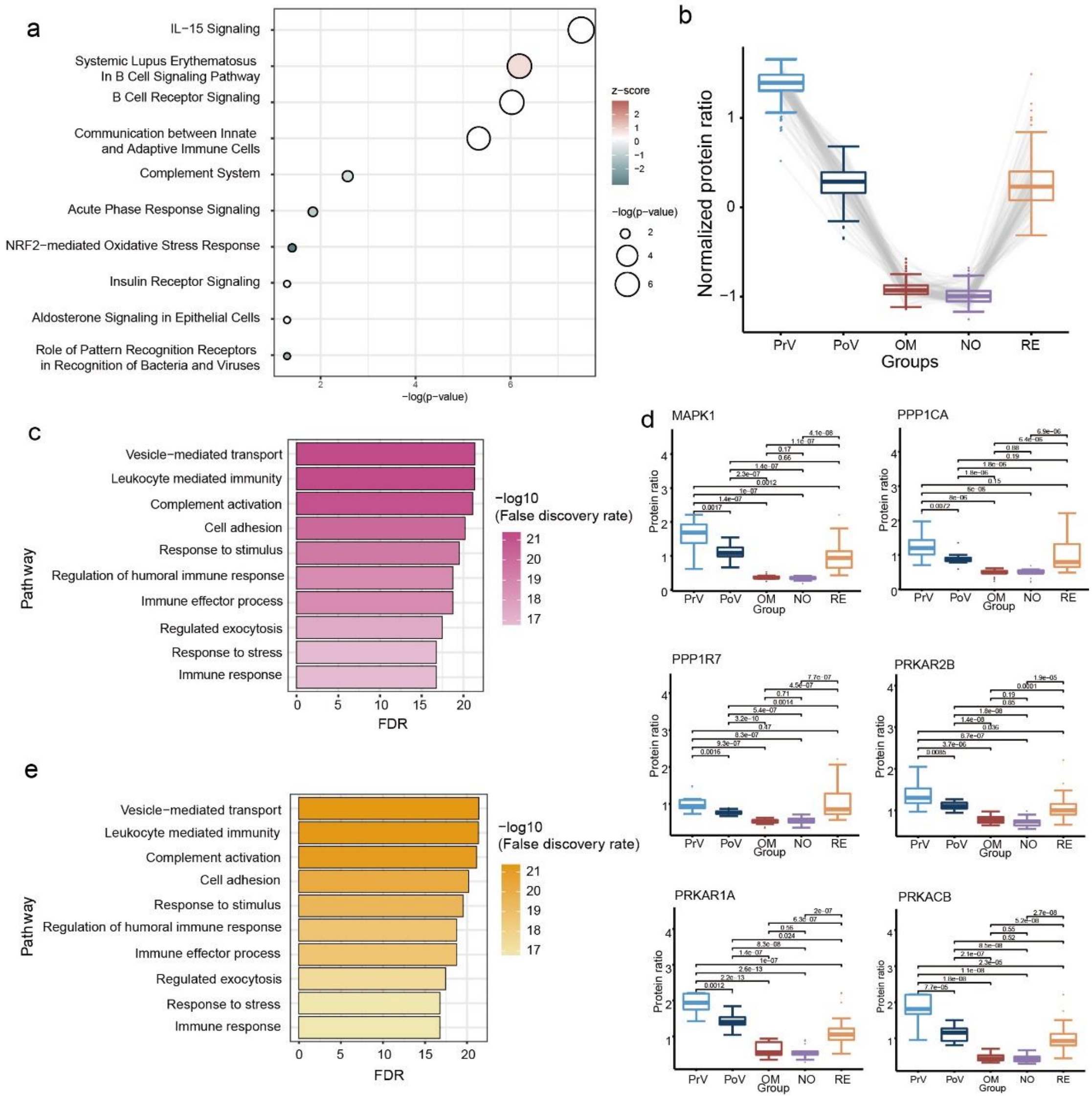

Supplementary Figure 2. Pathway enrichment of the differentially expressed proteins (DEPs). a Pathway enrichment of 514 DEPs (B-H adjusted $P < 0.05$, fold change > 1.2) between the Omicron and non-COVID-19 respiratory virus infections by IPA ($P < 0.05$). The color of the circle indicates the activation state of the pathways estimated by IPA. Red, activation state; green, suppression state. Transparent circle means that the state of the pathway cannot be estimated by IPA. **b** Boxplots showing the abundance of 107 downregulated DEPs (B-H adjusted $P < 0.05$, fold change > 1.2) in the post-vaccination samples compared with pre-vaccination samples in the five groups (PrV, PoV, OM, NO, and RE). Each gray line represents the median of one protein abundance. **c** Top ten pathways (ranking by false discovery rate (FDR), FDR < 0.05), biological processes of GO database, of the 107 downregulated DEPs. **d** Boxplots showing the expression of MAPK1, PPP1CA, PPP1R7, PRLAR2B, PRKAR1A, and PRKACB across the five groups (PrV, PoV, OM, NO, and RE). **e** Top ten pathways (ranking by FDR, FDR < 0.05), biological processes of GO database, of 406 DEPs (B-H adjusted $P < 0.5$, fold change > 1.2) in

the Omicron compared with post-vaccination samples. PrV, pre-vaccination; PoV, post-vaccination; OM, Omicron; NO, non-Omicron; RE, non-COVID-19 respiratory virus infection.

Supplementary Table 1. Clinical characteristics of the experimental cohort

Baseline Characteristics	Healthy (N=14)	Non-COVID-19 (N=29)	Omicron variant SARS-CoV-2 (N=17)	Other SARS-CoV-2 (N=7)	***P***
**Gender — no. [a](%)**					0.98
Male	3 (21.4)	17 (58.6)	9 (52.9)	4 (57.1)	
Female	11 (78.6)	12 (41.4)	8 (47.1)	3 (42.9)	
**Age — yr [b]**					0.91
Mean ± SD	26.1 ± 2.4	39.7 ± 14.4	42.82 ± 13.9	46 ± 20.5	
Median (IQR)	25 (25.0-26.75)	33 (30.0-25.0)	43 (31.0-49.0)	51 (36.0-60.5)	
Range	23-33	18-71	26-69	13-65	
**Infection severity — no. [a](%)**					0.54
Non-severe		26 (89.7)	17 (100)	7 (100)	
Severe		3 (10.3)	0	0	
**Comorbidities — no. (%)**					0.26
Hypertension		4 (13.8)	3 (17.6)	1 (14.3)	
Fatty liver disease (FLD)		0 (0.0)	2 (11.8)	0 (0.0)	
Osteoporosis		1 (3.4)	1 (5.9)	0 (0.0)	
Coronary heart disease (CHD)		2 (6.9)	1 (5.9)	0 (0.0)	
Dermatosis		7 (24.1)	0 (0.0)	0 (0.0)	
Others		8 (27.6)	3 (17.6)	1 (14.3)	
**Lymphocyte count, $\times 10^9$/L**					1
Mean ± SD		1.47 ± 0.83	1.29 ± 0.68	1.45 ± 0.54	
Median (IQR)		1.42 (0.80-1.64)	1.28 (0.77-1.66)	1.40 (1.04-1.77)	
Range		0.66-4.3	0.39-3.00	0.82-2.31	
**Leukocyte count, $\times 10^9$/L**					1
Mean ± SD		7.96 ± 4.55	5.41 ± 1.47	6.27± 2.33	
Median (IQR)		6.38 (5.24-8.43)	5.56 (4.36-6.59)	5.79 (4.62-7.66)	
Range		2.23-19.54	3.18-8.25	4.00-9.54	
**Monocyte count, $\times 10^9$/L**					1
Mean ± SD		0.68 ± 0.26	0.62 ± 0.25	0.50 ± 0.12	
Median (IQR)		0.61 (0.57-0.78)	0.58 (0.42-0.76)	0.49 (0.44-0.53)	
Range		0.27-1.31	0.35-1.39	0.34-0.72	
**Vaccinations — no. a(%)**					0.03
unvaccinated	0		1 (5.9)		
1st dose	0		2 (11.8)		
2nd dose	14 (100.0)		9 (52.9)		
3rd dose	0		2 (11.8)		
4th dose	0		2 (11.8)		
unknown	0		1 (5.9)		

[a] no.: number.

[b] yr.: year
